# Retraction: Prevalence and management of aphids (Hemiptera: Aphididae) in different wheat genotypes and their impact on yield and related traits

**DOI:** 10.1371/journal.pone.0272179

**Published:** 2022-08-03

**Authors:** The PLOS ONE Editors

The *PLOS ONE* Editors retract this article [1] because it was identified as one of a series of submissions for which we have concerns about authorship, competing interests, and peer review. We regret that the issues were not addressed prior to the article’s publication.

KZ, ON, SAA and MW either did not respond directly or could not be reached. FH, MAbbas, MArshad, SA, MF, AI, MJS, ATKZ, YL, and MJA did not agree with the retraction.
